# Bony Defect Regeneration in Periodontitis: A Systematic Review of the Literature Regarding the Use of Enamel Matrix Derivative Proteins

**DOI:** 10.3390/dj13030092

**Published:** 2025-02-20

**Authors:** Eugen Bud, Silvia-Izabella Pop, Anamaria Bud, Benjamin Robert Steele, Alexandru Vlasa

**Affiliations:** 1 Department of Orthodontics, Faculty of Dental Medicine, George Emil Palade University of Medicine, Pharmacy, Science, and Technology of Targu Mures, 540139 Târgu Mureș, Romania; eugen.bud@umfst.ro; 2 Department of Pedodontics, Faculty of Dental Medicine, George Emil Palade University of Medicine, Pharmacy, Science, and Technology of Targu Mures, 540139 Târgu Mureș, Romania; 3 Independent Researcher, 75012 Paris, France; benjamin18.steele@gmail.com; 4 Department of Periodontology and Oral-Dental Diagnosis, Faculty of Dental Medicine, George Emil Palade University of Medicine, Pharmacy, Science, and Technology of Targu Mures, 540139 Târgu Mureș, Romania; alexandru.vlasa@umfst.ro

**Keywords:** enamel matrix derivative proteins, bone regeneration, wall defects, periodontitis

## Abstract

**Background**: Periodontitis is characterized as a change in the total periodontal tissues that includes tissue loss, as evidenced by clinical loss of attachment, and radiographically determined alveolar bone loss, periodontal pockets, and gingival bleeding. **Objectives**: The aim of this study was to observe and analyze recent information from the literature on the effect of enamel matrix derivative proteins on the bony defects caused by periodontitis. **Methods**: Through using two major online databases and search engines, the literature was manually searched for papers published until May 2024. To find relevant studies, this research utilized a combination of target keywords, and the reference lists of manuscripts that were chosen for inclusion in this study were checked and analyzed in tabular form, enabling the collection and comparison of data. **Results**: According to the results, the average value of the probing depth gained was 4 mm for the EMD™ alone and 4.25 mm for the EMD combined with surgical techniques such as open-flap techniques, platelet derivatives, and growth factors. In regard to clinical attachment level (CAL) gaining, average values of 3.6 mm in EMD™ alone and 3.86 mm with EMD™ combined with other techniques were observed. **Conclusions**: It can be concluded that the healing propensity depends on the morphological structure of the bone defect represented by the wall stage, and there is a certain coherence and correlation between the values of probing depth (PD) and clinical attachment level (CAL), whether for the use of EMD alone or its use in combination with other materials.

## 1. Introduction

Periodontitis is characterized as a change in the total periodontal tissues that includes tissue loss, as evidenced by clinical loss of attachment, and radiographically determined alveolar bone loss, periodontal pockets, and gingival bleeding [1,2,3,4]. The new consensus opted on a classification model for periodontitis that is further defined by a multidimensional staging and grading system that may be updated when new data become available [5]. In accordance with the Chicago criterium, 2017, periodontitis, necrotic periodontitis, and periodontitis manifestations of systemic disorders are nowadays the three recognized types of periodontal diseases that impact the deep periodontium [5]. Bone loss can be due to different factors such as malocclusion, endodontic infections, trauma induced by prostheses, orthodontic treatments, and a lack of stimulation from the absence of an antagonist tooth [6,7].

Periodontal pockets induce the destruction of supporting periodontal tissues, leading to the loosening of the teeth and thereby bone loss. Two types of periodontal pockets can be differentiated [1,2,8,9,10]:

- Suprabony (supracrestal or supra-alveolar) pockets appear when the bottom of the pocket is coronal to the crest of the alveolar bone, and the pocket wall lies coronal to the bone. This type of bone loss is always horizontal.
- Intrabony (infrabony, subcrestal, or intra-alveolar) pockets appear when the bottom of the pocket is apical to the crest of the alveolar bone. With this second type, the lateral pocket wall lies between the tooth surface and the alveolar bone. The bone loss is in most cases vertical.

The most prevalent pattern of bone loss in periodontal disease is horizontal bone loss. The height of the bone is lowered, but the bone edge remains perpendicular to the tooth surface. The interdental septa, as well as the facial and lingual plates, are all damaged, but not to the same extent, around the same tooth. Bone loss can also be caused by misplaced teeth or partial or total edentation, which prevents correct chewing and hence deprives the bone of the necessary stimulation [11].

One of the most frequently commercial products available employed in the practice for periodontology treatment is called Emdogain^®^ (EMD) (Institute Straumann, Basel, Switzerland). Emdogain is a biological product consisting of a unique group of proteins extract of enamel matrix and contains amelogenins of various molecular weights. This gel represents a combination of freeze-dried DMA (powder) and a hydrogel (propylene glycol alginate) to complete the formulation [7,8,9,11,12,13]. This pure protein complex, freeze-dried and enriched with amelogenins, isolated from the amellar matrix collected from dental swine germs is referred to as a “enamel matrix derivative” (EMD) [13]. Amelogenins activate the proliferation and differentiation of periodontal fibroblasts and osteoblasts when absorbed on the root surface [7,9,12,13]. The regeneration of the periodontal ligament and cementum is the primary function of amelogenins in periodontal regeneration [14]. Amelogenins, present in EMD, represent an extracellular matrix protein complex that induce the formation of acellular cementa. The EMDs then promote the reconstruction of the periodontium in a complex mechanism of activation of osteo-regeneration through the bone cells while inhibiting the epithelialization of the damaged sites [5,6,7,8]. This mechanism is still relatively unknown, and studies are being conducted to understand these phenomena.

Periodontologists have been treating tissue abnormalities caused by periodontitis-related destruction of the tooth’s supporting tissues for many years. Periodontal disorders are complex infectious diseases with various inflammatory states [14]. The success of existing medicines in rebuilding the periodontium is thus a major challenge, and it has become a major public health concern. Although periodontal disease can be treated, irreversible tissue damage results. Various therapeutic options incorporating nonsurgical treatments, as well as conservative and regenerative surgical techniques, have been used for the treatment of intrabony defects throughout the last three decades, with varying degrees of success. The goal of regenerative periodontal therapy is to restore the lost periodontal structures such as the new formation of root cementa, periodontal ligaments, and alveolar bone. According to several studies, some periodontal lesions have been demonstrated to have bone regenerative capacity down to the remnant crest level. Several years ago, researchers, through their studies, came up with a new concept: that of tissue selection. The enamel matrix derivative, which is a combination of various proteins, can be used to illustrate this notion. The EMDs placed into the lesion trigger the induction, differentiation, and proliferation mechanisms, resulting in the regeneration of the tooth’s attachment system and, ultimately, bone defects; therefore, the null hypothesis of the present research was that there is significantly improved healing when comparing EMDs with conventional therapy. The aim of this study was to observe and analyze recent information published the literature on the effect of enamel matrix derivative proteins on the bony defects caused by periodontal disease.

## 2. Material and Methods

### 2.1. Search Method

Using the PubMed, Cochrane, and ResearchGate database, the literature was searched for papers published until May 2024. To find relevant studies, research utilized a combination of target MeSH keywords, text words, and subject headings. The authors then targeted two sets of keywords used in different combinations, as follows (Table 1):

➢ “Emdogain intrabony defects”;
➢ “Emdogain infrabony defects”.

Review article reference lists were manually searched, and in addition, the reference lists of articles that were chosen for inclusion in this study were checked and analyzed in tabular form to enable the authors to collect and compare the data.

### 2.2. Selection and Inclusion/Exclusion Criteria

Table 1 provides an overview of the main inclusion and exclusion criteria that were used and employed during the methodology. The studies were limited and filtered to human histological studies evaluating the effect of nonsurgical or surgical treatment for periodontal intrabony/infrabony defects, with or without the use of potentially regenerative materials.

Only articles written in English were included in this study. The regenerative material employed during the treatment procedure could include a combination of amelogenins and the following:

- barrier membranes;
- grafting materials;
- growth factors/proteins.

The authors chose the PICO framework in order to achieve the objectives of this study, since it is the most commonly used model for structuring clinical analyses and questions.

P (patient, problem)—human adult patients with no previous regenerative periodontal therapy in their medical history and no general disorders associated;

I (intervention, exposure)—periodontal regenerative therapy/bone regenerative therapy;

C (comparison)—EMD alone versus EMD plus barrier membranes/grafting materials/growth factors/proteins;

O (outcomes)—clinical attachment level, probing pocket depth, radiographic bone fill, or patient-reported outcomes at regular post-surgery check-ups.

### 2.3. Method of Screening

**Figure 1:**
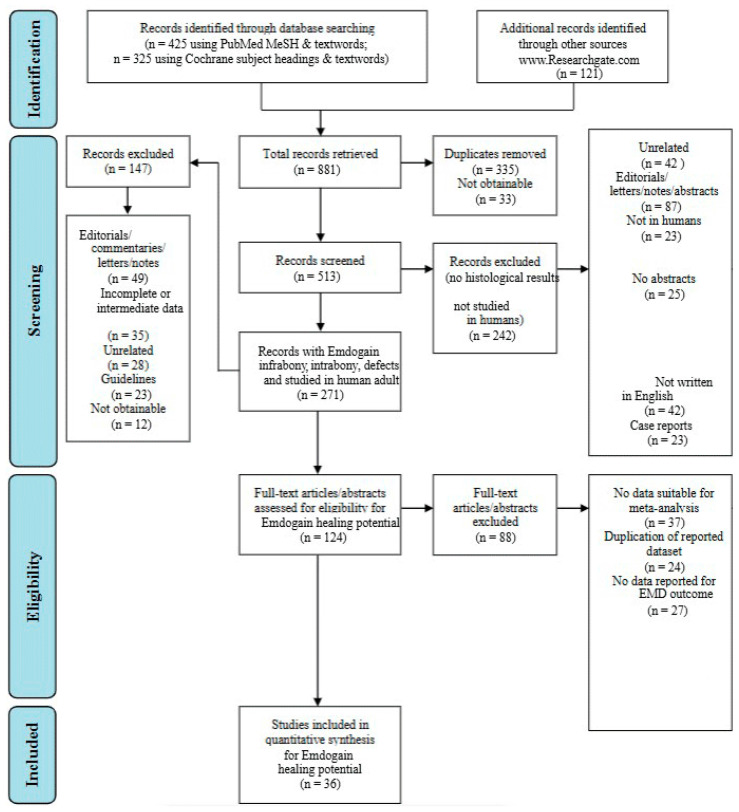
Flowchart regarding the methodology conducted within the study framework [15].

The selected studies’ titles and abstracts were independently screened. The main criterion employed to screen titles and abstracts was the following question tag: “Was the study conducted in people and did its findings present histological treatment outcomes in periodontal intrabony/infrabony lesions following the application of regenerative biomaterials?” If the screening question was answered “yes” or “uncertain”, the entire text of each item was collected. For the evaluation and quantification of the search conducted, the authors followed and complied the PRISMA statement guidelines as shown in Figure 1 [15].

The initial targeted research allowed the authors to find 881 articles in total. After filtering the research and duplicate removal, the authors were able to obtain 513 eligible papers. Following more filters applied to the search, as shown in Figure 1, the authors were able to retrieve a total of 124 papers, which were manually screened and assessed for eligibility. After eligibility was assessed, a total of 60 papers were discarded. Ultimately, a total of 36 papers were included in the present research.

**Figure 2:**
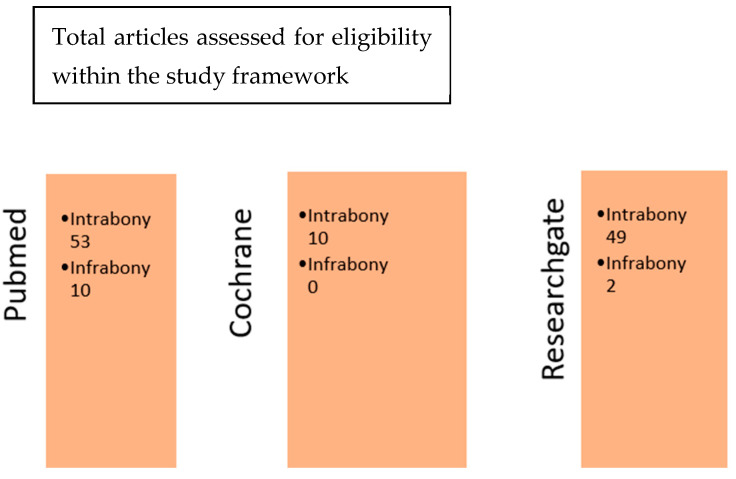
Cross-comparison of the three database search engines.

Later in this study, after refining our searches on the search engines PubMed, Cochrane, and ResearchGate, a cross-comparison according to the two sets of keywords used was performed. This cross-comparison between the respective sets of results obtained with the terms “Emdogain intrabony defects” and “Emdogain infrabony defects” allowed the authors to isolate the articles of interest for this study. By comparing the articles obtained from the three databases, the authors were able to see that some articles were either common to both or specific to both. The Cochrane database article collection contained 7 articles that were extremely specific, and they could not be found in the PubMed database (Figure 2). This specificity concerned mainly clinical research (see Table S1 in Supplementary Materials).

### 2.4. Data Extraction and Analysis

**Figure 3:**
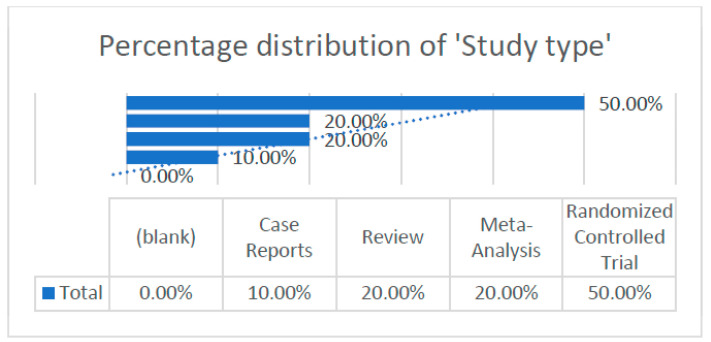
Percentage distribution of study type within the research framework.

According to the articles obtained, we were able to extract and analyze different data, which are illustrated in the following graph (Figure 3).

- Distribution of study types.

### 2.5. Risk of Bias and Quality Control

A quality assessment of the included studies was performed using the Revman Cochrane™ approach. The risk of bias tool identifies the domains specified in the Cochrane risk of bias instruments for systematic reviews. All the authors clearly defined both their study objective and the population (number, characteristics, and eligibility) on which they were going to carry out the research. Moreover, to assess the methodological quality of the included studies, the data from each article were independently evaluated by the authors using a structured manual form. The evaluation focused on key categories such as study design, primary outcomes related to oxidative stress and periodontal health, sample size, and study results.

## 3. Results

### Summary Table of Articles from the Search Engine Databases

After analyzing the articles collected [9,10,11,12,13,14,15,16,17,18,19,20,21,22,23,24,25,26,27,28,29,30,31,32,33,34,35,36,37,38,39,40,41,42,43,44,45,46,47,48,49,50,51,52,53,54,55,56,57,58,59,60,61,62] according to the keyword combinations intrabony/infrabony bone defects, the authors compiled a summary of the data in the following tables, which allowed a statistical analysis and discussion of the data inherent to Emdogain on several types of bone defects.

**Figure 4:**
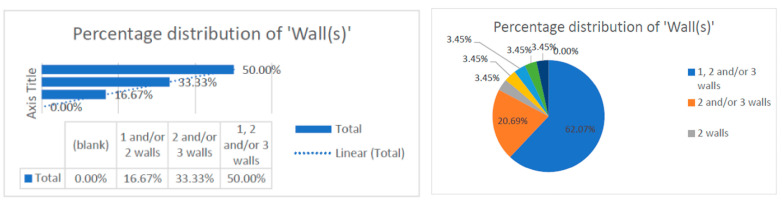
Distribution of the numbers of wall defects within the research.

This data collection [9,10,11,12,13,14,15,16,17,18,19,20,21,22,23,24,25,26,27,28,29,30,31,32,33,34,35,36,37,38,39,40,41,42,43,44,45,46,47,48,49,50,51,52,53,54,55,56,57,58,59,60,61,62] revealed that the number of treated defect walls per study included one, two, and/or three walls. The data were correlated with the type of study conducted, which was randomized. Most of the studies evaluated distribution of two and/or three walls (83.33%), and some studies conducted research on one-wall defects only (16.67%). This observation can be corelated with the technical difficulty of regenerating bone defects in only one wall and can be a reason for the reduced distribution of one-wall defects (16.67%) (Figure 4).

- Distribution of the numbers of walls
- Distribution of change in probing depth (PD).

**Figure 5:**
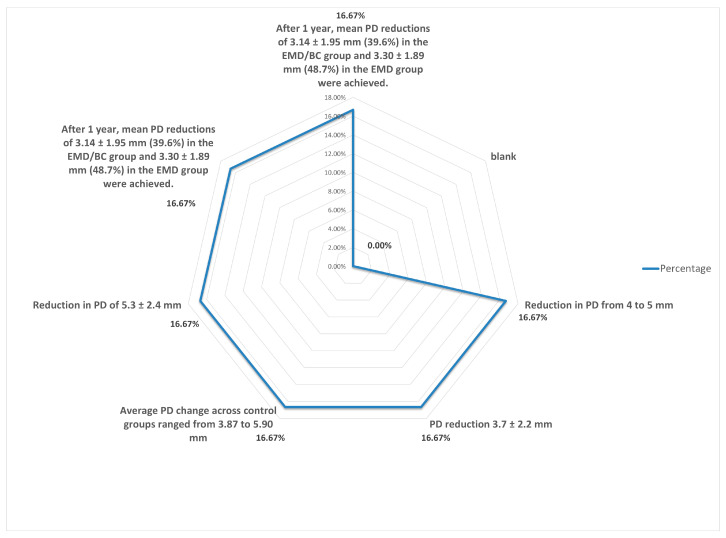
Distribution in change in probing depth.

**Figure 6:**
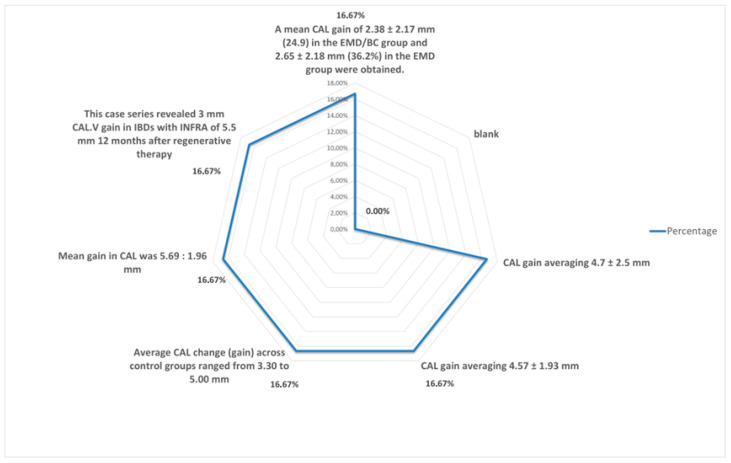
Percentage distribution of changes in probing depth.

Although it is statistically difficult to compare the values between methods due to the diversity of techniques, i.e., Emdogain combined with a membrane or with a growth factor, as shown in Table 2, Figure 5 and Figure 6, the different PD values and the techniques used revealed that the recorded average value of the probing depth gain is about 4 mm for the EMD alone and 4.25 mm for the EMD combined with different other techniques [9,10,11,12,13,14,15,16,17,18,19,20,21,22,23,24,25,26,27,28,29,30,31,32,33,34,35,36,37,38,39,40,41,42,43,44,45,46,47,48,49,50,51,52,53,54,55,56,57,58,59,60,61,62].

- Distribution of change in clinical attachment level (CAL).

**Figure 7:**
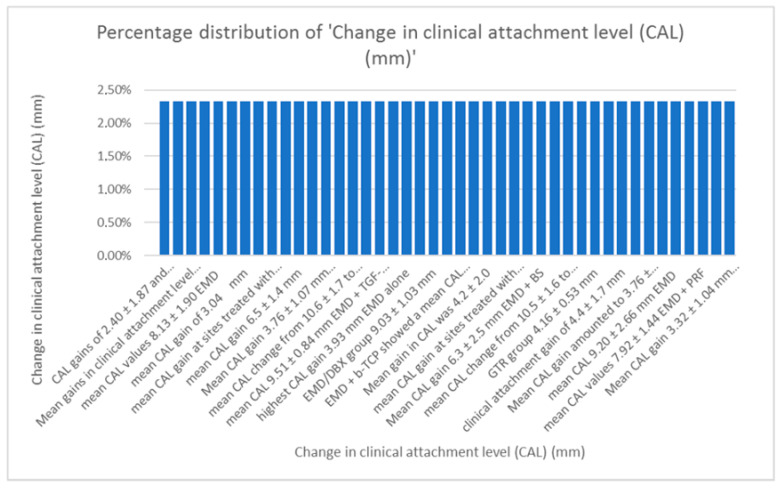
Distribution of changes in clinical attachment level.

When comparing data regarding the CAL, average values of 3.6 mm for the gain in CAL with EMD alone and 3.86 mm with EMD combined were calculated. As seen in Figure 7 data regarding CAL varies, as the techniques combined with Emdogain were numerous [9,10,11,12,13,14,15,16,17,18,19,20,21,22,23,24,25,26,27,28,29,30,31,32,33,34,35,36,37,38,39,40,41,42,43,44,45,46,47,48,49,50,51,52,53,54,55,56,57,58,59,60,61,62].

**Figure 8:**
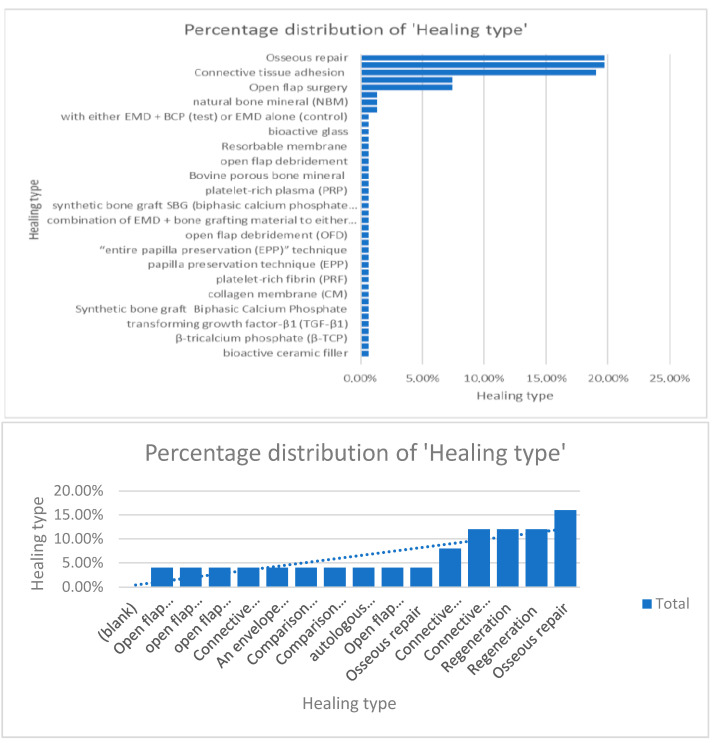
Percentage distribution of the surgical techniques used.

When comparing information regarding the percentage of distribution regarding the surgical protocol followed and healing type, the authors concluded that osseous repair, connective tissue adhesion, and open-flap surgery were the most used techniques [9,10,11,12,13,14,15,16,17,18,19,20,21,22,23,24,25,26,27,28,29,30,31,32,33,34,35,36,37,38,39,40,41,42,43,44,45,46,47,48,49,50,51,52,53,54,55,56,57,58,59,60,61,62] (Figure 8).

**Figure 9:**
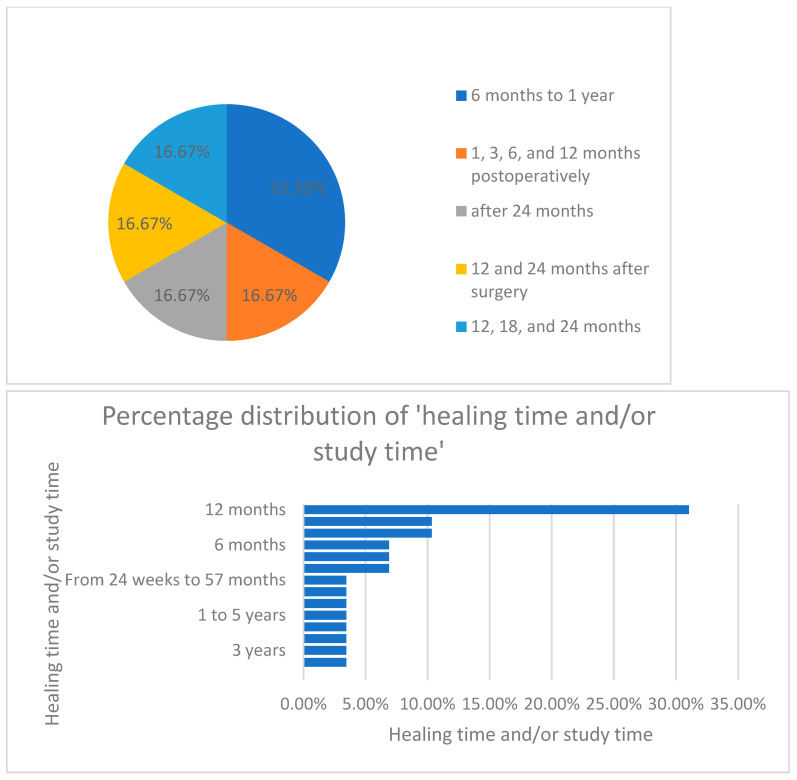
Percentage distribution of the healing time/study period.

Furthermore, when analyzing the distribution of healing time and study period, as seen in Figure 9, the authors concluded that most of the studies (33%) were conducted over a period of 12 months.

## 4. Discussion

Using a range of techniques and materials, periodontal regeneration in human intrabony defects can be accomplished to varying degrees, according to the findings of the current systematic study. The major goals of periodontal treatment are to eliminate infection and resolve chronic inflammation in order to stop its progression and prevent it from recurring. A lack of bleeding on probing and minimal probing pocket depths (less than 4 mm) are the clinical signs of this. Following the application of various bone grafts and analogs, guided tissue regeneration, biological agents, and other combinations, periodontal regeneration has been observed. Recent pioneering research showed that enamel matrix proteins might act as essential regenerative proteins capable of supporting periodontal regeneration, including the development of new cementa, functionally orientated periodontal ligament fibers, and new alveolar bone. There has been a good number of articles about the biological basis and therapeutic application of EMD.

Several interesting facts about clinical research have emerged. Sculean et al. [61] in his detailed observation of several studies has shown that, unexpectedly, the amount of periodontal regeneration, or new cementa with inserting fibers that are functionally orientated and new bone, was quite consistent across all treatments. Except for alloplastic substances and biological agents employed as monotherapies, which demonstrated little periodontal regeneration despite a wide range in intrabony defect depth prior to therapy, those findings were confirmed. A further interesting finding is that, in the majority of situations, the amount of newly formed cementa was either greater than or equal to the amount of newly formed bone and that wound stability and the provision of space are crucial for fully realizing the periodontum’s innate capacity for regeneration [61].

In the same way, clinical in vivo research revealed that even extremely extensive periodontal defects transplanted with a bone substitute by guided tissue regeneration exhibited complete regeneration given enough time for tissue remodeling and maturation. Therefore, the diminished bone growth seen in a number of biopsies may simply be the product of a short healing period [62].

A crucial factor that may lead to bias during the histopathological analysis and that must be taken into account for a fair interpretation of the healing outcome, and also for an equitable comparison between treatment modalities, is the variation in morphological characteristics and dimensions of naturally developing periodontal defects [63,64,65,66]. In fact, the vascular and cellular resources of the periodontal ligaments, alveolar bone, and gingiva that surround the defect appear to have a significant impact on the repair of deep three-walled intraosseous lesions and deep dehiscence or gingival recession defects. Contrarily, it is clear that in two- or one-walled intraosseous defects, the distribution and contribution of tissue resources are drastically changed and diminished [67,68,69]. In fact, the proportions of the defects seem to be a significant factor in predicting healing achievements in clinical settings, both after conventional surgical therapy. Where wide defects responded with less bone gain compared to narrow defects, and after periodontal regenerative surgery, better clinical outcomes, in other words, larger clinical attachment level (CAL) gain and bone fill, were achieved in deep, narrow intrabony defects compared to wide, shallow defects [63,64,65,66,67,68,69].

In line with our research, Miron et al. [21,38,57] pointed out the effects on early wound healing. All sites were re-evaluated using a visual analog scale to determine the level of post-treatment discomfort after a median of 4 weeks. The EMD administered had a beneficial impact on intraosseous defects, as shown by an evaluation of postoperative regeneration, healing, and morbidity. Regarding the clinical outcomes following intrabony defects using EMD alone, Miron et al. [21,38,57] highlighted in his clinical research and other several studies that EMD significantly improved CAL gains and pocket depths. These results were mainly achieved via an open-flap debridement (OFD) surgical technique.

When comparing the values, the authors were able to collect and statistically calculate those combinations including the guided tissue generation technique (GTR), barrier membranes, or grafting materials, and EMD combined with these different techniques and biomaterials acted in synergy and had a significant positive impact on bone regeneration, especially in horizontal bone loss. Vertical or angular defects affect the bone in an oblique manner, causing a hollowed-out depression alongside the root. The defect’s base is perpendicular to the surrounding bone, frequently resulting from the presence of an infrabony periodontal pocket; therefore, treatment success is more difficult.

Despite the fact that the enamel matrix derivative has been around for more than 20 years as a periodontal tissue regenerator [56,59,60], it is also astonishing that it is still one of the few biomaterials that can histologically show genuine periodontal regeneration with the production of new cementa, periodontal ligaments, and alveolar bone that is still readily available for clinical usage [63,64,65,66,67,68,69]. Future research is needed in order to validate the results of the present research, since the main limitation of the present study is the quite-low number of articles included in the methodology. Specific enamel matrix proteins have several biological functions, and more studies are being conducted to characterize how these activities affect the behavior of cells and tissues. From a clinical standpoint, it is also increasingly crucial to continue researching the use of EMDs to see if bone regeneration outcomes may be further enhanced by minor adjustments to EMD support systems or by minimally invasive surgical techniques. EMD continues to be one of the benchmarks for biologic-assisted periodontal regeneration.

## 5. Conclusions

The use of EMD alone or its use in combination with other materials (membranes, growth factors, etc.) and techniques offered reduced pocket depth, a gain in clinical attachment, and bone repairment. Through the present research concerning the use of EMD in relation to bone defects, the authors conclude that the healing propensity depends on the morphological structure of the bone defect represented by the wall stage and that there is a certain coherence and correlation between the values of probing depth (PD) and clinical attachment lost (CAL).

## Figures and Tables

**Table 1:** Inclusion and exclusion criteria within the study framework.

MeSH Keywords	Inclusion Criteria	Exclusion Criteria
Emdogain intrabony defects	Studies relevant to the review objectives: intrabony/infrabony defects	Studies involving animal subjects
Emdogain infrabony defects	Studies of adult patients covering all age groups	Seminar presentations/case reports and case studies
Bone regeneration	Randomized/non-randomized investigations and clinical trials	Opinion pieces
Tissue regeneration	Clinical cases of at least 10 patients	Incomplete or missing data
	Validated comparative analyses and reviews	Conference presentations and abstracts
	Papers written in English	Non-English-language articles

**Table 2:** Summary table for “Emdogain infrabony/intrabony defects”.

**Study Authors (Ref. No.)**	**Year of Publication**	**Study Type**	No. of Patients	No. of Defects	Wall(s)	Defect Depth (mm)	Healing Time and/or Study Time	Healing Type	Conclusions	Change in Probing Depth PD (mm)	Change in Clinical Attachment Level CAL (mm)
Pilloni et al. [9]	2014	Review	64 patients		1, 2, and/or 3 walls	Intrabony defect more than 3 mm on radiograph with PD ≥ 5 mm	12, 18, and 24 months	Regeneration Connective tissue adhesion Osseous repair Open-flap debridement + EMD	EMD + nano-HA synergic role	Reduction in PD of 5.3 ± 2.4 mm	CAL average gain of 4.7 mm ± 2.5 mm
Eickholz et al. [11]	2014	Randomized control trial	61 patients	57 defects	1, 2, and/or 3 walls	Infrabony defect depth ≥ 4 mm	12 and 24 months after surgery	Osseous repair Regeneration Open-flap Comparison EMD + Doxy versus EMD alone	200 mg Doxy for 7; no improvement in PD nor in CAL	PD reduction of 3.7 ± 2.2 mm	CAL gain of 4.7 ± 1.93 mm
Del Fabbro et al. [17]	2018	Meta-analysis		1402 defects	2 and/or 3 walls	Infrabony defect depth ≥ 3–6 mm	6 months to 1 year	APC + EMD vs. EMD Regeneration OFD Osseous repair	No advantages of APC + EMD	Average PD from 5.9 mm to 3.7 mm	
Ragghianti Zangrando et al. [18]	2014	Randomized control trial	10 patients	43 defects	2 and/or 3 walls	Infrabony defect ≥ 3 mm	After 24 months	OFD OFD + EMD Connective tissue adhesion	No clear results	Reduction of 4.21 ± 0.97 mm in PD average	CAL average gain of 5.69 ± 1.96 mm
Losada et al. [19]	2017	Randomized control trial	52 patients	46 defects	1 and/or 2 walls	Infrabony defect ≥ 3 mm	1, 3, 6, and 12 months after surgery	Regeneration Connective tissue adhesion Envelope full thickness flap	Statistically significant PD reduction and CAL gain	PD reduction of 3.14 ± 1.95 mm	CAL gain of 2.38 ± 2.17 mm
Nikcles et al. [20]	2017	Review	47 patients	41 defects	1, 2, and/or 3 walls		6 months to 1 year	Regeneration Osseous repair Connective tissue adhesion	Regenerative therapy had better results than OFD	Reduction in PD from 5 mm to 4 mm	3 mm CAL gain after 12 months
Miron RJ et al. [21]	2016	Review	434 patients		1, 2, and/or 3 walls	Infrabony defect ≥ 3–6 mm	6 to 12 months	Regeneration OFD Osseous repair	Histological genuine regeneration	Mean PD reduction of 4.22 ± 1.2 mm	CAL gain of 3.76 ± 1.07 mm
Fileto Mazzonetto et al. [22]	2021	Randomized control trial	100 patients	60 defects	1, 2, and/or 3 walls	Infrabony defect ≥ 3–6 mm	12 months	Simplified papilla preservation flap Regeneration Osseous repair	Statistically significant PD reduction and CAL gain	Mean PD reduction of 4.22 ± 1.2 mm	CAL gain of 3.76 ± 1.07 mm
Machot et al. [22]	2014	Randomized control trial	38 patients		1, 2, and/or 3 walls	Width ≥ 2 mm and depth ≥ 4 mm	6 to 12 months	EMD + NHA Regeneration	More studies are required to prove potential advantages	PD reduction from 5 mm to 4 mm	CAL gain of 4.2 mm ± 2 mm
Rojas et al. [25]	2019	Randomized control trial	199 patients	220 defects	1, 2, and/or 3 walls	Infrabony defect ≥ 3 mm deep	12 months	Comparison EMD vs. GTR	No clear advantages	PD reduction of 3.4 ± 1.2 mm	CAL gain of 4.4 ± 1.7 mm
Matarasso et al. [28]	2015	Review	434 patients	548 defects	1, 2, and/or 3 walls	Infrabony defect ≥ 3 mm deep	6, 8, and 12 months	EMD ± bone graft	EMD + bone graft better results than EMD alone	Mean PD reduction of 4.22 ± 1.2 mm	CAL gain of 3.76 ± 1.07 mm
Corbella et al. [29]	2019	Randomized control trial	9 patients	20 defects	2 walls		12 months	EMD + bovine-derived bone substitutes	No clear results	PD reduction of 3 ± 0.7 mm	CAL gain of 6.9 ± 1.1 mm
Aslan et al. [30]	2020	Randomized control trial	30 patients	52 defects	1, 2, and/or 3 walls		12 months	EMD + bovine-derived bone substitutes	No statistically significant results	PD reduction of 6.5 ± 2.65 mm	CAL gain of 6.3 ± 2.5 mm
Pietruska et al. [31]	2012	Randomized control trial	24 patients		1, 2, and/or 3 walls		12 months	EMD+ BCP vs. EMD alone	No clear results	PD reduction of 4.7 ± 0.8 mm	CAL gain of 10.4 ± 1.3 mm
Koop et al. [32]	2012	Review			1, 2, and/or 3 walls	Intrabony defect 3 to 4 mm deep	12 months	Bovine porous bone mineral Bioactive glass EMD Bioactive ceramic filler	EMD more effective than controls	EMD superior to OFD with 1.52 mm	CAL gain compared to control: 1.3 mm
Dori et al. [34]	2013	Randomized control trial	22 patients	32 defects	2 and/or 3 wall defects		12 months	EMD + natural bone mineral/ β TCP	Results can be sustained over 10 years		
Iorio-Siciliano et al. [35]	2014	Randomized control trial	40 patients	40 defects	1, 2, and/or 3 walls	Intrabony depth ≥3 mm	12 months	EMD + deproteinized bovine bone mineral/ collagen membrane	Similar results at 12 months		
Parashis et al. [36]	2012	Randomized control trial	61 patients	61 defects	2 and/or 3 walls	Intrabony depth ≥ 4 mm	12 months	OFD Regeneration Osseous repair	Statistically significant improvement	PD reduction of 3.9 ± 1 mm	CAL gain of 6.5 ± 1.4 mm
Mueller et al. [12]	2013	Meta-analysis			1, 2, and/or 3 walls	Intrabony depth ≥ 3 mm	12 months	Regeneration Connective tissue adhesion Osseous repair	Significant results when using EMD	Mean PD reduction of 4.05 mm	Mean CAL gain of 3.04 mm
Bertoldi et al. [39]	2019	Review	22 patients	42 defects	1, 2, and/or 3 walls	Intrabony depth ≥ 3 mm	12 months	Regeneration Connective tissue adhesion Osseous repair	Significant results when using EMD at 1 and 2 years	PD reduction of 3.3 mm at 1 year and 3.4 mm at 2 years	CAL gain of 2.9 mm at 1 and 2 years
Hoffmann et al. [40]	2016	Randomized control trial	30 patients	30 defects	1, 2, and/or 3 walls	Intrabony depth ≥ 3 mm	6, 12, and 36 months	Regeneration EMD ± BCP	No discernible benefit	PD reduction of 3.93 mm	CAL gain of 3.8 ± 2.2 mm
Trombelli et al. [41]	2021	Meta-analysis	10 patients/study	10 defects/study	1, 2, and/or 3 walls	Intrabony depth ≥ 3 mm	12 months	Bone graft Regeneration Osseous repair	EMD + bone graft shows more discernible results than EMD alone	Residual PD EMD + graft 4.32 mm	CAL gain from 3.65 to 4.1 mm
Dori et al. [43]	2013	Randomized control trial	24 patients	36 defects			3, 6, and 12 months	EMD+ natural bone mineral/ Platelet-rich plasma	PRP does not appear to improve results	PD reduction of 8.7 ± 1.7 mm	CAL from 10.5 ± 1.6 mm to 6 ± 1.7 mm
Agrali et al. [44]	2016	Randomized control trial	12 patients	30 defects	2 and/or 3 walls	Intrabony depth ≥ 3 mm	6 months	EMD + autogenous bone graft/ transforming growth factor TGF-β1	Significant improvement	PD reduction of 3.2 mm	CAL gain of 9.51 ± 0.84 mm
Mitani et al. [46]	2015	Comparative study	40 patients	43 defects	2 and/or 3 walls	Intrabony depth ≥ 3 mm	1, 3, and 5 years	GTR OFD	EMD + GTR improved results compared to OFD	PD reduction of 2.8 mm	CAL gain of 6.3 ± 1.3 mm
Aydemir Turkal et al. [47]	2016	Randomized control trial	28 patients	56 defects	1, 2 and/or 3 walls		6 months	Platelet-rich fibrin OFD Connective tissue adhesion	Effective in intrabony defects	PD reduction of 2.50 ± 0.51 mm for EMD + PRF	CAL gain of 7.93 ± 1.44 mm for EMD + PRF
Anoixiadou et al. [48]	2022	Randomized control trial	34 patients	38 defects		Intrabony defects ≤ 7 mm	6 and 12 months	Minimal invasive nonsurgical technique	EMD usage does not increase radiological and clinical results	PD reduction of 4.2 mm in MINST + EMD	CAL gain of 3.5 ± 1.4 mm
Ogihara et el. [49]	2014	Randomized control trial	69 patients		1, 2 and/or 3 walls		6 and 12 months	Freeze-dried bone allograft Demineralized freeze-dried bone allograft	Significant results in profound defects	PD reduction of 4.2 mm	CAL gain of 4.1 mm
Aslan et al. [50]	2017	Case reports	12 patients	12 defects	2 and/or 3 walls	Intrabony defects ≥ 7 mm	6 to 12 months	EMD + entire papilla preservation Regeneration	Significant results in interproximal defects	PD reduction of 7 ± 2.8 mm	CAL gain of 6.83 ± 2.51 mm
Bhutda et al. [51]	2013	Randomized control trial	15 patients	30 defects	2 and/or 3 walls		1 to 5 years	EMD + OFD Osseous repair Bone repair	Considerable reduction in PD and CAL gain	At 5 years, PD reduction was 3.40 ± 0.57	At 5 years, mean CAL was 4.9 ± 1.91 mm
De Leonardis et al. [56]	2013	Randomized control trial	34 patients	72 defects	1 and/or 2 walls	Intrabony depth ≥ 3 mm	12 and 24 months	EMD + HA/ β-TCP	Improved clinical and radiographical results	PD reduction of 4.0 ± 0.42 mm	CAL gain of 3.47 ± 0.65 mm
Aydemir Turkal et al. [47]	2016	Randomized control trial	28 patients	56 defects	1 and/or 2 walls		6 months	EMD + platelet-rich fibrin	Beneficial result in intrabony defects	PD reduction of 2.71 ± 0.75 mm	CAL value of 8.13 ± 1.9 mm
Mikami et al. [59]	2022	Comparative study	151 patients	253 defects	1 and/or 2 or 3 walls		3 years	Periodontal regenerative therapy (PRT) Connective tissue adhesion	PRT + EMD greatly improved clinical results over time	PD reduction of 2.84 ± 1.73 mm	CAL gain of 2.4 ± 1.84 mm1
Artzi et al. [60]	2015	Comparative study	32 patients	32 defects	1 and/or 2 or 3 walls		12 months	Guided tissue regeneration Deproteinized bone xenograft particle (DBX)	Good clinical outcomes in aggressive periodontitis		CAL gain of 9.03 ± 1.03 mm for EMD + DBX
Sculean et al. [61]	2015	Review	118 patients		1 and/or 2 or 3 walls	Infrabony defects of 3 to 6 mm	From 24 weeks to 57 months	Regeneration Bone graft Long junctional epithelium	Significant improvement when using EMD		
Stavropoulos et al. [62]	2021	Meta-analysis	634 patients	573 defects	1 and/or 2 or 3 walls	Infrabony defects from 3 to 7 mm	24 to 36 months	GTR DBX Resorbable membranes	Significant improvements when using EMD		

## Supplementary Materials

The following supporting information can be downloaded at https://www.mdpi.com/article/10.3390/dj13030092/s1, Table S1: PRISMA guideline checklist; original tables regarding the methodology conducted.
