# Supplementary material for: Bony Defect Regeneration in Periodontitis: A Systematic Review of the Literature Regarding the Use of Enamel Matrix Derivative Proteins
Source: Dent J (Basel). 2025 Feb 20;13(3):92. doi: 10.3390/dj13030092 (PMC11941161; doi:10.3390/dj13030092)
Supplement: Supplementary file 1 [file dentistry-13-00092-s001.zip › dentistry-3467113-supplementary.pdf]

PRISMA 2020 Checklist

Section and Topic	Item #	Checklist item	Location where item is reported
**TITLE**			
Title	1	Identify the report as a systematic review.	Page 1, title
**ABSTRACT**			
Abstract	2	See the PRISMA 2020 for Abstracts checklist.	Page 2
**INTRODUCTION**			
Rationale	3	Describe the rationale for the review in the context of existing knowledge.	Page 2, lines 51-60
Objectives	4	Provide an explicit statement of the objective(s) or question(s) the review addresses.	Page 3, lines 103-107
**METHODS**			
Eligibility criteria	5	Specify the inclusion and exclusion criteria for the review and how studies were grouped for the syntheses.	Page 3, lines 130-134
Information sources	6	Specify all databases, registers, websites, organisations, reference lists and other sources searched or consulted to identify studies. Specify the date when each source was last searched or consulted.	Page 3, lines 110-113
Search strategy	7	Present the full search strategies for all databases, registers and websites, including any filters and limits used.	Page 4, lines 134-141
Selection process	8	Specify the methods used to decide whether a study met the inclusion criteria of the review, including how many reviewers screened each record and each report retrieved, whether they worked independently, and if applicable, details of automation tools used in the process.	Page 5, lines 145-150
Data collection process	9	Specify the methods used to collect data from reports, including how many reviewers collected data from each report, whether they worked independently, any processes for obtaining or confirming data from study investigators, and if applicable, details of automation tools used in the process.	Page 5, lines 150-153
Data items	10a	List and define all outcomes for which data were sought. Specify whether all results that were compatible with each outcome domain in each study were sought (e.g. for all measures, time points, analyses), and if not, the methods used to decide which results to collect.	Page 5, lines 155-160
	10b	List and define all other variables for which data were sought (e.g. participant and intervention characteristics, funding sources). Describe any assumptions made about any missing or unclear information.	Page 6, lines 161-166, figure 3
Study risk of bias assessment	11	Specify the methods used to assess risk of bias in the included studies, including details of the tool(s) used, how many reviewers assessed each study and whether they worked independently, and if applicable, details of automation tools used in the process.	Pages 6-7, lines 170-178
Effect measures	12	Specify for each outcome the effect measure(s) (e.g. risk ratio, mean difference) used in the synthesis or presentation of results.	Pages 6-7, lines 170-178
Synthesis	13a	Describe the processes used to decide which studies were eligible for each synthesis (e.g. tabulating the study intervention characteristics and	Page 6,

Section and Topic	Item #	Checklist item	Location where item is reported
methods		comparing against the planned groups for each synthesis (item #5)).	lines 163-165
	13b	Describe any methods required to prepare the data for presentation or synthesis, such as handling of missing summary statistics, or data conversions.	Page 6, lines 150-160
	13c	Describe any methods used to tabulate or visually display results of individual studies and syntheses.	Page 6, lines 1630165
	13d	Describe any methods used to synthesize results and provide a rationale for the choice(s). If meta-analysis was performed, describe the model(s), method(s) to identify the presence and extent of statistical heterogeneity, and software package(s) used.	Page 6, line 140-145
	13e	Describe any methods used to explore possible causes of heterogeneity among study results (e.g. subgroup analysis, meta-regression).	-
	13f	Describe any sensitivity analyses conducted to assess robustness of the synthesized results.	-
Reporting bias assessment	14	Describe any methods used to assess risk of bias due to missing results in a synthesis (arising from reporting biases).	Pages 6-7, lines 170-178
Certainty assessment	15	Describe any methods used to assess certainty (or confidence) in the body of evidence for an outcome.	Pages 6-7, lines 170-178
**RESULTS**			
Study selection	16a	Describe the results of the search and selection process, from the number of records identified in the search to the number of studies included in the review, ideally using a flow diagram.	Figure 1, page 5
	16b	Cite studies that might appear to meet the inclusion criteria, but which were excluded, and explain why they were excluded.	Suppl. File 2
Study characteristics	17	Cite each included study and present its characteristics.	Table 2
Risk of bias in studies	18	Present assessments of risk of bias for each included study.	Table 2
Results of individual studies	19	For all outcomes, present, for each study: (a) summary statistics for each group (where appropriate) and (b) an effect estimate and its precision (e.g. confidence/credible interval), ideally using structured tables or plots.	Table 2
Results of syntheses	20a	For each synthesis, briefly summarise the characteristics and risk of bias among contributing studies.	Table 2
	20b	Present results of all statistical syntheses conducted. If meta-analysis was done, present for each the summary estimate and its precision (e.g. confidence/credible interval) and measures of statistical heterogeneity. If comparing groups, describe the direction of the effect.	-
	20c	Present results of all investigations of possible causes of heterogeneity among study results.	Table 2
	20d	Present results of all sensitivity analyses conducted to assess the robustness of the synthesized results.	-
Reporting biases	21	Present assessments of risk of bias due to missing results (arising from reporting biases) for each synthesis assessed.	Page 14, lines 193-197
Certainty of	22	Present assessments of certainty (or confidence) in the body of evidence for each outcome assessed.	Page 14,

PRISMA 2020 Checklist

Section and Topic	Item #	Checklist item	Location where item is reported
evidence			lines 193-197
**DISCUSSION**			
Discussion	23a	Provide a general interpretation of the results in the context of other evidence.	Page 17, lines 245-255
	23b	Discuss any limitations of the evidence included in the review.	Page 18, lines 293-301
	23c	Discuss any limitations of the review processes used.	Page 18, lines 302-305
	23d	Discuss implications of the results for practice, policy, and future research.	Page 18, lines 305-311
**OTHER INFORMATION**			
Registration and protocol	24a	Provide registration information for the review, including register name and registration number, or state that the review was not registered.	Page 19
	24b	Indicate where the review protocol can be accessed, or state that a protocol was not prepared.	Page 19
	24c	Describe and explain any amendments to information provided at registration or in the protocol.	Page 19
Support	25	Describe sources of financial or non-financial support for the review, and the role of the funders or sponsors in the review.	Page 19
Competing interests	26	Declare any competing interests of review authors.	Page 19
Availability of data, code and other materials	27	Report which of the following are publicly available and where they can be found: template data collection forms; data extracted from included studies; data used for all analyses; analytic code; any other materials used in the review.	Page 19

From: Page MJ, McKenzie JE, Bossuyt PM, Boutron I, Hoffmann TC, Mulrow CD, et al. The PRISMA 2020 statement: an updated guideline for reporting systematic reviews. BMJ 2021;372:n71. doi: 10.1136/bmj.n71.
This work is licensed under CC BY 4.0. To view a copy of this license, visit https://creativecommons.org/licenses/by/4.0/

Study: authors (ref. no.)	Year	Study type	No. of patients	No. of defects	Wall(s)	Defect depth (mm)	Healing time &/or study time	Healing type	Histological results	Change in probing depth (PD) (mm)	Change in clinical attachment level (CAL) (mm)	Source
Pilloni & Al.	2014	Review	64 patients		1, 2 and/or 3 walls	Infrabony defect more than 3 mm deep on radiographs, with pocket depth (PD) ≥5 mm	12, 18, and 24 months	Regeneration<br>Connective tisse adhesion<br>Osseous repair<br>open flap debridement, application of enamel matrix derivatives (EMD), nanohydroxyapatite (nanoHA) application, and combined nanoHA and EMD application	In summary, within the limitations of this study, we found that EMD and nanoHA played a synergic role in promoting restoration of the tooth-supporting apparatus. The group that received EMD+HA showed greater mean reduction in PD (5.75 mm) and improvement in gingival recession compared with the EMD only group.	Reduction in PD of 5.3 ± 2.4 mm,	CAL gain averaging 4.7 ± 2.5 mm	BioMed Research International/Hindawi Publishing Corporation
Rasperini & Al.	2013	Randomized Controlled Trial	No access to the article									Offical Journal of The Academy of Osseointegration
Eickholz P & Al.	2014	Randomized Controlled Trial	61 patients	57 defects	1, 2 and/or 3 walls	Infrabony defect depth ≥4 mm	12 and 24 months after surgery	Regeneration<br>Connective tissue adhesion<br>Osseous repair<br>Open flap surgery<br>Comparison between (EMD+Doxycyclin) and EMD	200 mg systemic DOXY for 7 days after regenerative therapy of infrabony defects using EMD failed to result in better bone fill, PD reduction, and CAL V gain	PD reduction 3.7 ± 2.2 mm	CAL gain averaging 4.57 ± 1.93 mm	Journal of Periodontology
Mueller & Al.	2013	Meta-Analysis	Information not related to our topic									Journal of Periodontal Research
Ghezzi & Al.	2016	Randomized Controlled Trial	No access to the article									Offical Journal of The Academy of Osseointegration
Fung & Al.	2012	Case Reports	Information not related to our topic									Journal of Clinical Orthodontics

Study: authors (ref. no.)	Year	Study type	No. of patients	No. of defects	Wall(s)	Defect depth (mm)	Healing time &/or study time	Healing type	Histological results	Change in probing depth (PD) (mm)	Change in clinical attachment level (CAL) (mm)	Source
Del Fabbro & Al.	2018	Meta-Analysis		1402 defects	2 and/or 3 walls	Infrabony defect ≥ (3-6) mm deep	6 months to 1 year	Comparison (APC + EMD) versus EMD<br>Regeneration<br>Connective tissue adhesion<br>Osseous repair<br>Open flap debridement (OFD)<br>autologous platelet concentrates (APC)	For GTR or EMD, insufficient evidence of an advantage in using APC was observed.	Average PD change (gain) across control groups ranged from 3.87 to 5.90 mm	Average CAL change (gain) across control groups ranged from 3.30 to 5.00 mm	Cochrane Meta Analysis
Ragghianti Zangrando & Al.	2014	Randomized Controlled Trial	10 patients	43 defects	2 and/or 3 walls	Infrabony defect >3 mm	after 24 months	open flap debridement (OFD) or OFD associated with enamel matrix derivative<br>Regeneration<br>Connective tissue adhesion	Linear radiographic analysis was not able to demonstrate superiority of EMD treated infrabony defects when compared to ODF after 24 months	reduction in PPD of 4.21 ± 0.97 mm	Mean gain in CAL was 5.69 ± 1.96 mm	BMC Oral Health
Losada M & Al.	2017	Randomized Controlled Trial	52 patients	46 defects	1 and/or 2 walls	Infrabony defect >3 mm	1, 3, 6, and 12 months postoperatively	Regeneration<br>Connective tissue adhesion<br>Osseous repair<br>An envelope full-thickness mucoperiosteal flap was raised buccally and lingually following simplified papilla preservation incisions	The results of the present study demonstrated favorable outcomes with both EMD and the combination therapy of EMD and BC after 12 months. Both therapies resulted in statistically significant PD reduction, CAL gain, and bone fill of non contained infrabony defects.	After 1 year, mean PD reductions of 3.14 ± 1.95 mm (39.6%) in the EMD/BC group and 3.30 ± 1.89 mm (48.7%) in the EMD group were achieved.	A mean CAL gain of 2.38 ± 2.17 mm (24.9%) in the EMD/BC group and 2.65 ± 2.18 mm (36.2%) in the EMD group were obtained.	Journal of Periodontology
Nickles & Al.	2017	Review	47 patients	41 defects	1, 2 and/or 3 walls		6 months to 1 year	Regeneration<br>Osseous repair<br>Connective tissue adhesion	After intracrevicular incision, a mucoperiosteal flap was raised to a height of 5 mm, exposing the bony margin of the defect and allowing complete visualization of the infrabony lesion<br>In infrabony defects (IBDs), regenerative therapy achieves superior clinical results compared with open flap debridement. This applies equally for the use of barrier membranes and enamel matrix derivative (EMD)<br>CAL values achieved by regenerative therapy may have retained stability over 5 years	Reduction in PD from 4 to 5 mm	This case series revealed 3 mm CAL V gain in IBDs with INFRA of 5.5 mm 12 months after regenerative therapy	Journal of Periodontology

Study: authors (ref. no.)	Year	Study type	No. of patients	No. of defects	Wall(s)	Defect depth (mm)	Healing time &/or study time	Healing type	Histological results	Change in probing depth (PD) (mm)	Change in clinical attachment level (CAL) (mm)	Source
Miron RJ & Al.	2016	Review	434 patients		1, 2 and/or 3 walls	intrabony defect ≥ [3-6] mm deep	6 to 12 months	combination of EMD + bone grafting material to either EMD alone or bone grafting material alone<br>Regeneration<br>Connective tissue adhesion<br>Osseous repair<br>Open flap surgery	EMD is still one of the few biomaterials that can histologically demonstrate genuine periodontal regeneration, including cementum creation, periodontal ligament production, and alveolar tissue regeneration.	Mean PD reduction measured 4.22 ± 1.20 mm at sites treated with EMD and bone graft and yielded 4.12 ± 1.07 at sites treated with EMD alone	Mean CAL gain amounted to 3.76 ± 1.07 mm following treatment with EMD + bone graft and to 3.32 ± 1.04 mm following treatment with EMD alone	Journal of Clinical Periodontology
Szatmári P. & Al.	2014	Miscellaneous							Article non available			Fogorv Sz.
Fileto Mazzonetto & Al.	2021	Randomized Controlled Trial	100 patients	60 defects	1, 2 and/or 3 walls	intrabony defect ≥ [3-6] mm deep	12 months	Following simplified papilla preservation flap (SPPF) surgery.<br>Regeneration<br>Connective tissue adhesion<br>Osseous repair				Journal of Periodontology
Al Machot & Al.	2014	Randomized Controlled Trial	38 patients		1 and/or 2 walls	wide (>2 mm) and deep (≥4 mm)	6 to 12 months	comparison using either an enamel matrix derivative (EMD) or a nanocrystalline hydroxyapatite (NHA)<br>Regeneration<br>Connective tissue adhesion<br>Osseous repair<br>Open flap surgery	studies with larger sample size are needed to prove a potential advantage of EMD compared to NHA	Reduction in PD from 4 to 5 mm	Mean gain in CAL was 4.2 ± 2.0	Biomed Research International
Rojas MA & Al.	2019	Randomized Controlled Trial	199 patients	220 defects	1, 2 and/or 3 walls	intrabony defect ≥ 3 mm deep	12 months	Comparison between EMD and GTR<br>Regeneration<br>Connective tissue adhesion<br>Osseous repair<br>Open flap surgery	It is unable to prove that the EMD has a clear favorable influence on early wound healing outcomes after surgical treatment of periodontal intrabony defects.	PPD reductions of 3.4 ± 1.2 mm	clinical attachment gain of 4.4 ± 1.7 mm	BMC Oral Health
Stavropoulos & Al.	2021	Randomized Controlled Trial							Article not relevant to our topic			Dental Clinics of North America
Gupta SJ & Al.	2014	Randomized Controlled Trial							Article not relevant to our topic			Journal of the International Academy of Periodontology
Matarasso M & Al.	2015	Review	434 patients	548 defects	1, 2 and/or 3 walls	infrabony defect ≥ 3 mm deep	6, 8 and 12 months	combination of enamel matrix derivative (EMD) and bone graft compared with that of EMD alone<br>Regeneration<br>Connective tissue adhesion<br>Osseous repair<br>Open flap surgery<br>Bone graft	The combination of EMD and bone grafts may result in additional clinical improvements in terms of CAL gain and PD reduction compared with those obtained with EMD alone	Mean PD reduction measured 4.22 ± 1.20 mm at sites treated with EMD and bone graft<br>Mean PD reduction measured 4.12 ± 1.07 mm at sites treated with EMD alone	Mean CAL gain 3.76 ± 1.07 mm following treatment with a combination of EMD and bone graft<br>Mean CAL gain 3.32 ± 1.04 mm (median 3.40; 95 % CI 3.28–3.52) following treatment with EMD alone	Clinical Oral Investigation
Corbella & Al.	2019	Randomized Controlled Trial	9 patients	20 defects	2 walls		12 months	enamel matrix derivative (EMD) alone or in association with deproteinized bovine bone<br>Regeneration<br>Connective tissue adhesion<br>Osseous repair<br>Open flap surgery	EMD alone and the use of a combination of EMD and DBBM for the treatment of partially contained defects showed comparable clinical and radiographic outcomes after 12 months	Mean PD reduction 2.8 ± 0.8 mm EMD alone<br>Mean PD reduction reduced 3.0 ± 0.7 mm for EMD and DBBM	Mean CAL gain 5.0 ± 2.8 mm EMD alone<br>Mean CAL gain 6.9 ± 1.1 mm EMD and DBBM	Australian Dental Journal
Aslan S & Al.	2020	Randomized Controlled Trial	30 patients	52 defects	1, 2 and/or 3 walls		12 months	enamel matrix proteins plus bovine-derived bone substitutes (EPP EMD + BS)<br>papilla preservation technique (EPP)<br>Regeneration<br>Connective tissue adhesion<br>Osseous repair	No statistically significant differences were detected	Mean PD reduction 6.5 ± 2.65 mm EMD + BS<br>Mean PD reduction 6.2 ± 1.33 mm EMD alone	Mean CAL gain 6.3 ± 2.5 mm EMD + BS<br>CAL gain 5.83 ± 1.12 mm EMD alone	Journal of Clinical Periodontology

Study: authors (ref. no.)	Year	Study type	No. of patients	No. of defects	Wall(s)	Defect depth (mm)	Healing time &/or study time	Healing type	Histological results	Change in probing depth (PD) (mm)	Change in clinical attachment level (CAL) (mm)	Source
Pietruska M & Al.	2012	Randomized Controlled Trial	24 patients		1, 2 and/or 3 walls		12 months	Synthetic bone graft Biphasic Calcium Phosphate with either EMD + BCP (test) or EMD alone (control)<br>Regeneration<br>Connective tissue adhesion<br>Osseous repair	No differences in any of the investigated parameters were observed at baseline between the two groups	Mean PD reduction 4.4 ± 0.8 mm EMD<br>Mean PD reduction 4.7 ± 0.8 mm EMD + BCP	CAL gain from 10.4 ± 1.3 mm to 6.9 ± 1.0 mm EMD<br>CAL gain from 10.8 ± 1.6 mm to 7.4 ± 1.6 mm EMD + BCP	Clinical Oral Investigations
Gupta SJ & Al.	2014	Randomized Controlled Trial							Article not available			Journal of the International Academy of Periodontology
Koop & Al.	2012	Review			1, 2 and/or 3 walls	intrabony defect depth from 3 to 4 mm	12 months	Bone graft<br>Bovine porous bone mineral<br>bioactive glass<br>bioactive ceramic filler<br>Regeneration<br>Connective tissue adhesion<br>Osseous repair<br>Resorbable membrane	EMD is more effective than control therapies in treating intrabony defects, but not as effective as resorbable membranes. When EMD is combined with a coronally advanced flap for recession coverage, the results are better than a control, but not as good as a connective tissue transplant.	EMD was superior to OFD, with a mean difference of 1.52 mm<br>EMD was superior to placebo, with a mean difference of 0.48 mm<br>EMD was superior to EDTA, with a mean difference of 0.60 mm<br>EMD was superior to OFD/EDTA/placebo, with a mean difference of 0.92 mm<br>there was no statistically significant difference between EMD versus RM (0.03 mm	CAL gain for EMD 1.30 mm compared with the control treatments (OFD/EDTA/placebo)	Journal of Periodontology
Liu W & Al.	2012	Review							article not available			Acta Odontologica Scandinavica
Döri F & Al.	2013	Randomized Controlled Trial	22 patients	32 defects	2 and/or 3 walls		12 months	natural bone mineral (NBM)<br>β-tricalcium phosphate (β-TCP)<br>Regeneration<br>Connective tissue adhesion<br>Osseous repair	the current findings suggest that clinical benefits achieved with regenerative surgery utilizing EMD + NBM or EMD + -TCP can be sustained over a 10-year period.		EMD + NBM demonstrated a mean CAL change from 8.9 – 1.5 mm to 5.3 – 0.9 mm<br>EMD + b-TCP showed a mean CAL change from 9.1 – 1.6 mm to 5.4 – 1.1 mm	Journal of Periodontology
Iorio-Siciliano V & Al.	2014	Randomized Controlled Trial	40 patients	40 defects	1, 2 and/or 3 walls	intrabony depth ≥3 mm	12 months	deproteinized bovine bone mineral (DBBM)<br>collagen membrane (CM)<br>Regeneration<br>Connective tissue adhesion<br>Osseous repair	After 12 months, regeneration treatment utilizing either EMD + DBBM or CM + DBBM had similar clinical outcomes in profound non-contained intrabony lesions.	Reduction in PD 8.2 ± 1.9 mm EMD + DBBM<br>Reduction in PD 8.1 ± 2.1 mm EMD + CM	mean CAL gain at sites treated with EMD + DBBM 3.8 ± 1.5 mm<br>mean CAL gain at sites treated with CM + DBBM 3.7 ± 1.2 mm	Journal of Periodontology
Parashis AO & Al.	2012	Randomized Controlled Trial	61 patient	61 defects	2 and/or 3 walls	intrabony defect depth ≥4 mm	12 months	Regeneration<br>Connective tissue adhesion<br>Osseous repair<br>open flap surgery	the present results confirms that EMD treatment of intrabony defects can result in significant improvements (CAL gain, PD reduction, and defect resolution)	Reduction in PD 3.9 ±1.0 mm	mean CAL gain 6.5 ± 1.4 mm	Journal of Periodontology
Ferrarotti F & Al.	2018	Review							Data non available			Int J Periodontics Restorative Dent

Study: authors (ref. no.)	Year	Study type	No. of patients	No. of defects	Wall(s)	Defect depth (mm)	Healing time &/or study time	Healing type	Histological results	Change in probing depth (PD) (mm)	Change in clinical attachment level (CAL) (mm)	Source
Mueller VT & Al.	2013	Meta-Analysis			1, 2 and/or 3 walls	intrabony defect depth ≥3 mm	12 months	Regeneration<br>Connective tissue adhesion<br>Osseous repair<br>open flap surgery	Using EMD to address periodontal infrabony defects during open flap surgery was found to be beneficial in both research periods when compared to groups who did not use EMD.	mean probing pocket depth reduction of 4.05 mm	mean CAL gain of 3.04 mm	Journal of Periodontal Research
Bertoldi C & Al.	2019	Review							Article non available			Journal of Biological Regulators and Homeostatic Agents
Seshima F & Al.	2017	Review	22 patients	42 defects	1, 2 and/or 3 walls	intrabony defect of ≥3 mm in depth	12 and 24 months	Regeneration<br>Connective tissue adhesion<br>Osseous repair<br>open flap surgery	EMD treatment resulted in statistically significant increases in CAL and decreases in PD.	mean probing pocket depth reduction of 3.3 and 3.4 mm at 1 and 2 years	Mean gains in clinical attachment level (CAL) at 1 and 2 years were 2.9 mm	BMC research note
Hoffmann T & Al.	2016	Randomized Controlled Trial	30 patients	30 defects	1, 2 and/or 3 walls	intrabony defect depth ≥3 mm	6, 12 and 36 months	Regeneration<br>Connective tissue adhesion<br>Osseous repair<br>synthetic bone graft SBG (biphasic calcium phosphate BCP)	When comparing the usage of EMD with SBG to the use of EMD alone, there was no discernible benefit.	PPD reductions of 3.88 mm EMD +SBG<br>PPD reductions of 3.93 mm EMD	A mean gain in clinical attachment of 4.1 mm (±3.6) EMD + SBG<br>A mean gain in clinical attachment of 3.8 mm (±2.2) EMD	Clinical Oral Investigations
Trombelli L & Al.	2021	Meta-Analysis	10 patients/study	10 defects/study	1, 2 and/or 3 walls	intrabony defect depth ≥3 mm	12 months	Regeneration<br>Connective tissue adhesion<br>Osseous repair<br>Bone graft<br>Open flap surgery	When comparing the usage of EMD with a Graft to the use of EMD alone, there was discernible benefit.	highest residual probing depth for EMD 4.08 mm<br>highest residual probing depth for EMD + graft 4.32 mm	highest CAL gain 3.93 mm EMD alone<br>CAL gain ranged from 3.65 mm to 4.10 mm EMD + Graft	JDR Clinical & Translational Research
Liu Y & Al.	2017	Meta-Analysis			2 and/or 3 walls	intrabony defect depth ≥3 mm		Regeneration<br>Connective tissue adhesion<br>Osseous repair<br>Bone graft<br>Open flap surgery	Article not relevant to our topic			Acta Odontologica Scandavinica
Döri F & Al.	2013	Randomized Controlled Trial	24 patients	36 defects			3, 6, 12 months	natural bone mineral (NBM)<br>platelet-rich plasma (PRP)<br>Regeneration<br>Connective tissue adhesion<br>Osseous repair<br>Bone graft	the use of PRP does not appear to improve the results obtained with EMD + NBM	PPD reductions of 8.7 ± 1.7 mm EMD + NBM + PRP<br>PPD reductions of 8.8 ± 2.0 mm EMD + NBM	mean CAL change from 10.5 ± 1.6 to 6.0 ± 1.7 mm EMD + NBM + PRP<br>mean CAL change from 10.6 ± 1.7 to 6.1 ± 1.5 mm EMD + NBM	Journal of Periodontology
Agrali ÖB & Al.	2016	Randomized Controlled Trial	12 patients	30 defects	2 and/or 3 walls	intrabony defect depth ≥3 mm	6 months	autogenous bone graft (ABG)<br>transforming growth factor-β1 (TGF-β1)<br>open flap debridement (OFD)<br>Regeneration<br>Connective tissue adhesion<br>Osseous repair<br>Bone graft	All treatment procedures led to significant improvements	PPD reductions of 3.22 ± 0.41 mm EMD + TGF-β1<br>PPD reductions of 3.30 ± 0.67 mm EMD	mean CAL 9.51 ± 0.84 mm EMD + TGF-β1<br>mean CAL 9.20 ± 2.66 mm EMD	Nigerian Journal of Clinical Practice
Aimetti M & Al.	2016	Review							Article not available			Clinical Oral Investigations
Mitani A & Al.	2015	Comparative Study	40 patients	43 defects	2 and/or 3 walls	intrabony defect depth ≥3 mm	1, 3 and 5 years	guided tissue regeneration (GTR)<br>open-flap debridement (OFD)<br>Regeneration<br>Connective tissue adhesion<br>Osseous repair	The findings imply that periodontal surgery utilizing EMD and GTR techniques resulted in significantly higher clinical attachment levels and bone filling than OFD alone.	PPD reductions of 1.0 ± 0.3 mm OFD<br>PPD reductions of 2.8 ± 0.4 mm GTR<br>PPD reductions of 1.7 ± 0.3 mm EMD	A mean gain in clinical attachment of 6.3 ± 1.3 mm OFD<br>A mean gain in clinical attachment of 7.8 ± 1.8 mm GTR<br>A mean gain in clinical attachment of 6.9 ± 2.6 mm EMD	Journal of Periodontal Research

Study: authors (ref. no.)	Year	Study type	No. of patients	No. of defects	Wall(s)	Defect depth (mm)	Healing time &/or study time	Healing type	Histological results	Change in probing depth (PD) (mm)	Change in clinical attachment level (CAL) (mm)	Source
Aydemir Turkal H & Al.	2016	Randomized Controlled Trial	28 patients	56 defects	1, 2 and/or 3 walls		6 months	platelet-rich fibrin (PRF)<br>Open flap surgery<br>Regeneration<br>Connective tissue adhesion<br>Osseous repair	it can be concluded that both modalities are beneficial in intrabony defect treatment	PPD reductions of 2.71 ± 0.75 mm EMD<br>PPD reductions of 2.50 ± 0.51 mm EMD + PFR	mean CAL values 8.13 ± 1.90 EMD<br>mean CAL values 7.92 ± 1.44 EMD + PRF	Journal of Clinical Periodontology
Anoixiadou S & Al.	2022	Randomized Controlled Trial	34 patients	18 defects	3 walls	intrabony defects ≤7 mm	6 and 12 months	minimally invasive non-surgical technique (MINST)<br>Regeneration<br>Connective tissue adhesion<br>Osseous repair	The inclusion of EMD does not increase the mean clinical or radiographic results any further.	PD reduction 4.2 ± 1.7 mm MINST+EMD<br>PD reduction 4.0 ± 1.4 mm MINST	CAL gain 3.5 ± 1.4 mm MINST+EMD<br>CAL gain 3.4 ± 1.6 mm MINST	Journal of Clinical Periodontology
Ogihara S & Al.	2014	Randomized Controlled Trial	69 patients		1, 2 and/or 3 walls		6 and 12 months	freeze-dried bone allograft (FDBA)<br>demineralized FDBA (DFDBA)<br>Regeneration<br>Connective tissue adhesion<br>Osseous repair	When coupled with EMD, both graft materials played great in treating profound intrabony defects.	PD reduction 4.4 mm, (4.0 to 4.7) EMD/FDBA<br>PD reduction 3.7 mm, (3.4 to 4.0) EMD/DFDBA<br>PD reduction 3.3 mm, (3.0 to 3.6) EMD	CAL gain 4.1 mm, (3.8 to 4.5) EMD/FDBA<br>CAL gain 3.5 mm, (3.0 to 4.0) EMD/DFDBA<br>CAL gain 3.0 mm, (2.5 to 3.6) EMD	Journal of Periodontology
Aslan S & Al.	2017	Case Reports	12 patients	12 defects	2 and/or 3 walls	intrabony defect with probing depth (PD) ≥7 mm	6 to 12 months	"entire papilla preservation (EPP)" technique<br>Regeneration<br>Connective tissue adhesion<br>Osseous repair	The "EPP" is a new surgical method that allows access to deep interproximal intrabony defects while maintaining the integrity of the defect-associated interdental papilla.	PD reduction 7±2.8 mm	CAL gain of 6.83±2.51 mm	Journal of Clinical Periodontology
Bhutda G & Al.	2013	Randomized Controlled Trial	15 patients	30 defects	2 and/or 3 walls		1 to 5 years	open flap debridement<br>Regeneration<br>Connective tissue adhesion<br>Osseous repair<br>Bone graft	The application of Emdogain® with OFD with results in a considerable reduction in probing pocket depth (PPD) and gains in clinical attachment level (CAL) over a long-term follow-up, according to the data.	mean probing pocket depth (PPD) reduced from 7.24 ± 1.11 mm at baseline to 3.12 ± 0.87 mm at 1 year. At 5 years it was found to be 3.40 ± 0.57 mm.	1 year post-surgery, the mean clinical attachment level (CAL) was 4.12 ± 0.81 mm as compared to 8.08 ± 1.34 at baseline. Five years after surgery, mean CAL was found to be 4.90 ± 1.21 mm.	Acta Odontologica Scandavinica
Apicella A & Al.	2017	Miscellaneous	Article not relevant to our topic									Journal of Periodontology
Aimetti M & Al.	2017	Randomized Controlled Trial	Article not relevant to our topic									Clinical Oral Investigations
Wang Y & Al.	2016	Miscellaneous	Article not relevant to our topic									Journal of Periodontal Research
Nemoto Y & Al.	2018	Comparative Study	Article not available									Journal of Periodontal Research
De Leonardis D & Al.	2013	Randomized Controlled Trial	34 patients	72 defects	1 and/or 2 walls	intrabony defect depth ≥3 mm	12 and 24 months	open-flap debridement (OFD)<br>hydroxyapatite and β-tricalcium phosphate (HA/β-TCP) implant	Our findings support the idea that using a HA/-TCP composite implant in combination with EMD can improve the clinical and radiographic results of surgical treatment of unfavorable intrabony defects.	At 12 and 24 months after treatment, the EMD + HA/β-TCP group showed significantly greater PD reduction (4.00 ± 0.42 mm; 4.25 ± 0.63 mm)	At 12 and 24 months after treatment CAL gain (3.47 ± 0.65 mm; 3.63 ±0.91 mm)	Journal of Periodontology

Study: authors (ref. no.)	Year	Study type	No. of patients	No. of defects	Wall(s)	Defect depth (mm)	Healing time &/or study time	Healing type	Histological results	Change in probing depth (PD) (mm)	Change in clinical attachment level (CAL) (mm)	Source
Aydemir Turkal H & Al.	2016	Randomized Controlled Trial	28 patients	56 defects	1, 2 and/or 3 walls		6 months	platelet-rich fibrin (PRF)	it can be concluded that both modalities are beneficial in intrabony	PPD reductions of 2.71 ± 0.75 mm EMD	mean CAL values 8.13 ± 1.90 EMD	Journal of Clinical Periodontology
Miron RJ & Al.	2013	Randomized Controlled Trial	Article not relevant to our topic									Journal of Clinical Periodontology
de Sanctis M & Al.	2013	Comparative Study	Article not available									Int J Periodontics Restorative Dent
Mikami R & Al.	2022	Comparative Study	151 patients	253 defects	1, 2 and/or 3 walls		3 years	Regeneration<br>Connective tissue adhesion<br>Osseous repair<br>Bone graft<br>periodontal regenerative therapy (PRT)	Regardless of the patient's age, PRT with EMD greatly improved clinical results over time.	PPD reductions of 2.84 ± 1.73 and 2.87 ± 1.87 mm	CAL gains of 2.40 ± 1.87 and 2.47 ± 1.89 mm	Journal of Clinical Periodontology
Artzi Z & Al.	2015	Comparative Study	32 patients	32 defects	1, 2 and/or 3 walls		12 months	guided tissue regeneration (GTR) using deproteinized bone xenograft (DBX) particles and a resorbable membrane (the GTR group), or an application of enamel matrix derivatives (EMD) combined with DBX (the EMD/DBX group).<br>Regeneration<br>Connective tissue adhesion<br>Osseous repair<br>Bone graft	At 1-year post-treatment, surgical treatment of Aggressive periodontitis patients with GTR or EMD/DBX produced similar good clinical outcomes.	GTR group 3.58 ± 0.50 mm<br>EMD/DBX group 8.93 ± 1.14 mm	GTR group 4.16 ± 0.53 mm<br>EMD/DBX group 9.03 ± 1.03 mm	Journal of Clinical Periodontology
Sculean & Al.	2015	Review	118 patients		1, 2 and/or 3 walls	intrabony defects from 3 to 6 mm	From 24 weeks to 57 months	Long junctional epithelium<br>Connective tissue adhesion<br>Osseous repair<br>Bone graft<br>Regeneration	All treatment procedures led to significant improvements with the use of EMD			Periodontology 2000
Stavropoulos & Al.	2021	Meta-Analysis	634 patiens	573 defects	1, 2 and/or 3 walls	intrabony defects from 3 to 7 mm	From 24 to 36 months	guided tissue regeneration (GTR) using deproteinized bone xenograft (DBX) particles and a resorbable membrane (the GTR group), or an application of enamel matrix derivatives (EMD) combined with DBX (the EMD/DBX group).<br>Regeneration<br>Connective tissue adhesion<br>Osseous repair<br>Bone graft	All treatment procedures led to significant improvements with the use of EMD			Journal of Clinical Periodontology
